# Supplementary material for: An Integrated Multi-Omics Analysis Identifying Immune Subtypes of Pancreatic Cancer
Source: Int J Mol Sci. 2023 Dec 21;25(1):142. doi: 10.3390/ijms25010142 (PMC10779306; doi:10.3390/ijms25010142)

Fig. S1. External validation dataset (GSE62452) was utilized to validate clustering algorithms. (A) K = 3 was the optimal suggested value of number of clusters. (B) Visualisation of the cluster results for GEO samples. (C) Survival analysis for PC patients among clusters. (D) Silhouette plot for the PC clusters. (E) Consensus map of NMF clustering.

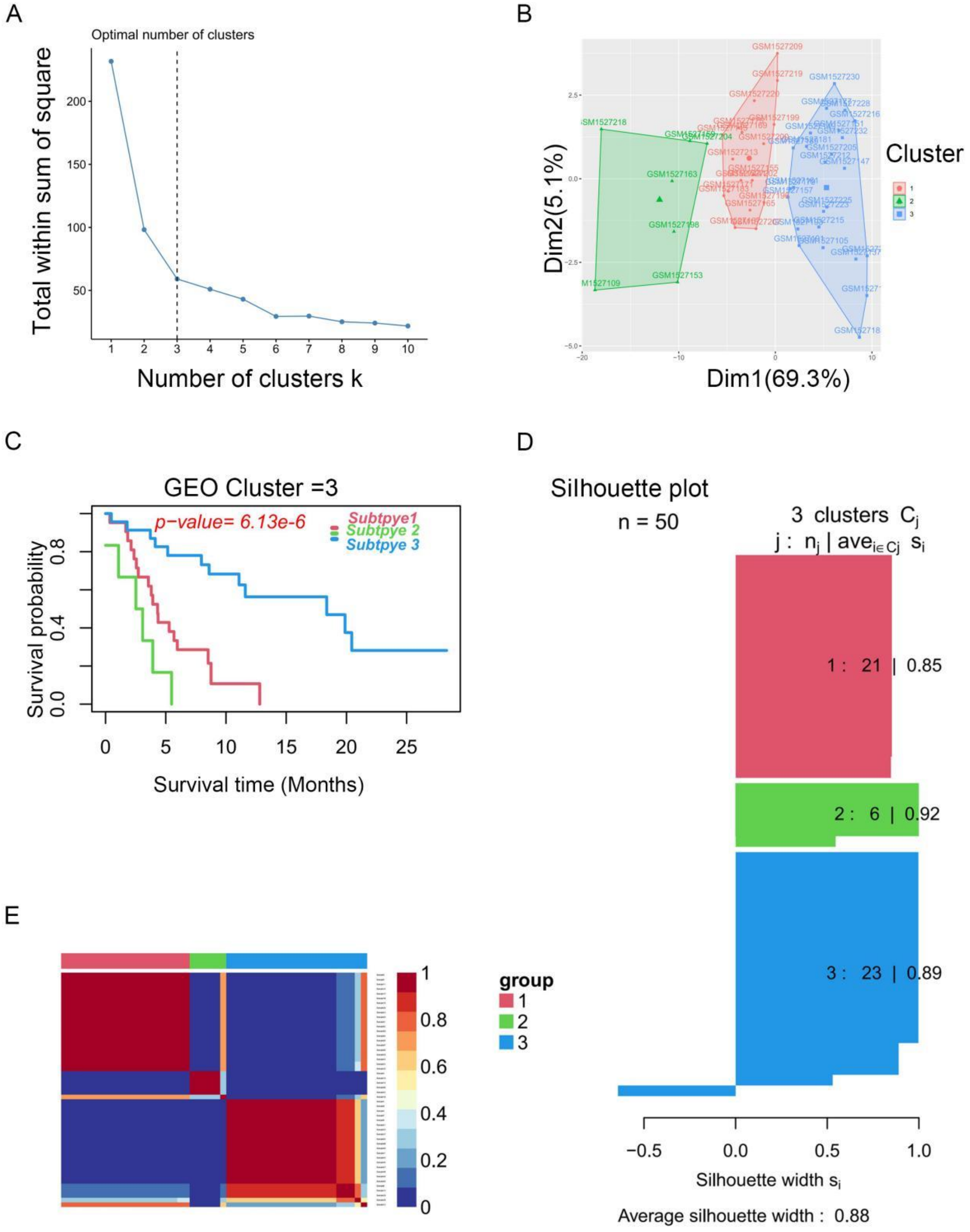

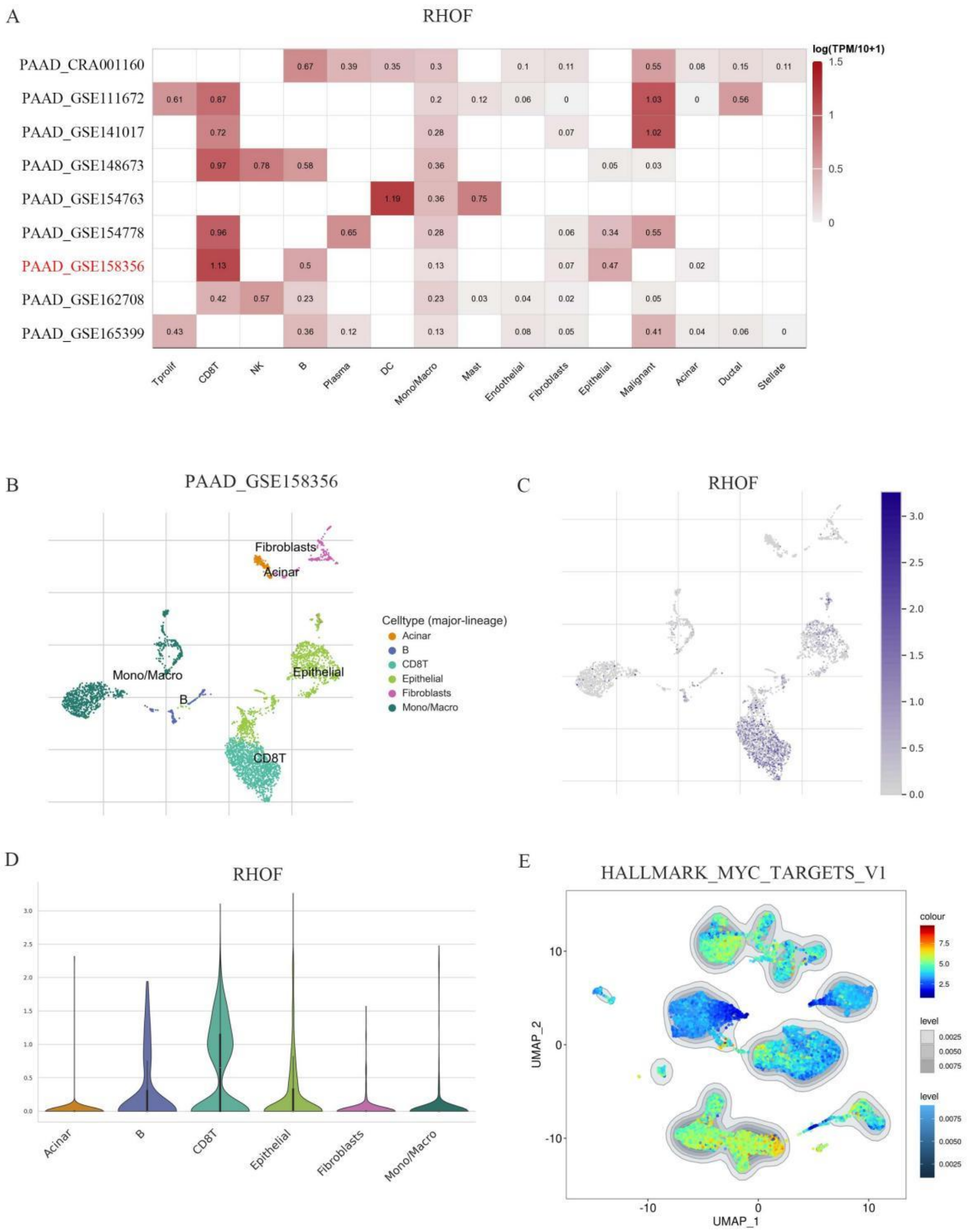

Fig. S2. The single-cell RNA sequencing analysis exhibits the expression pattern as well as the signal pathway of RHOF. (A) Summary of RHOF expression of 15 cell types in 9 single cell datasets. (B) The UMAP projection of cell subpopulations of PAAD-GSE158356 dataset from TISCH database. (C-D) RHOF expression from PAAD-GSE158356; (E) GSEA showed the the activity of the hallmark MYC targets V1 pathway in different cells based on TISCH database.

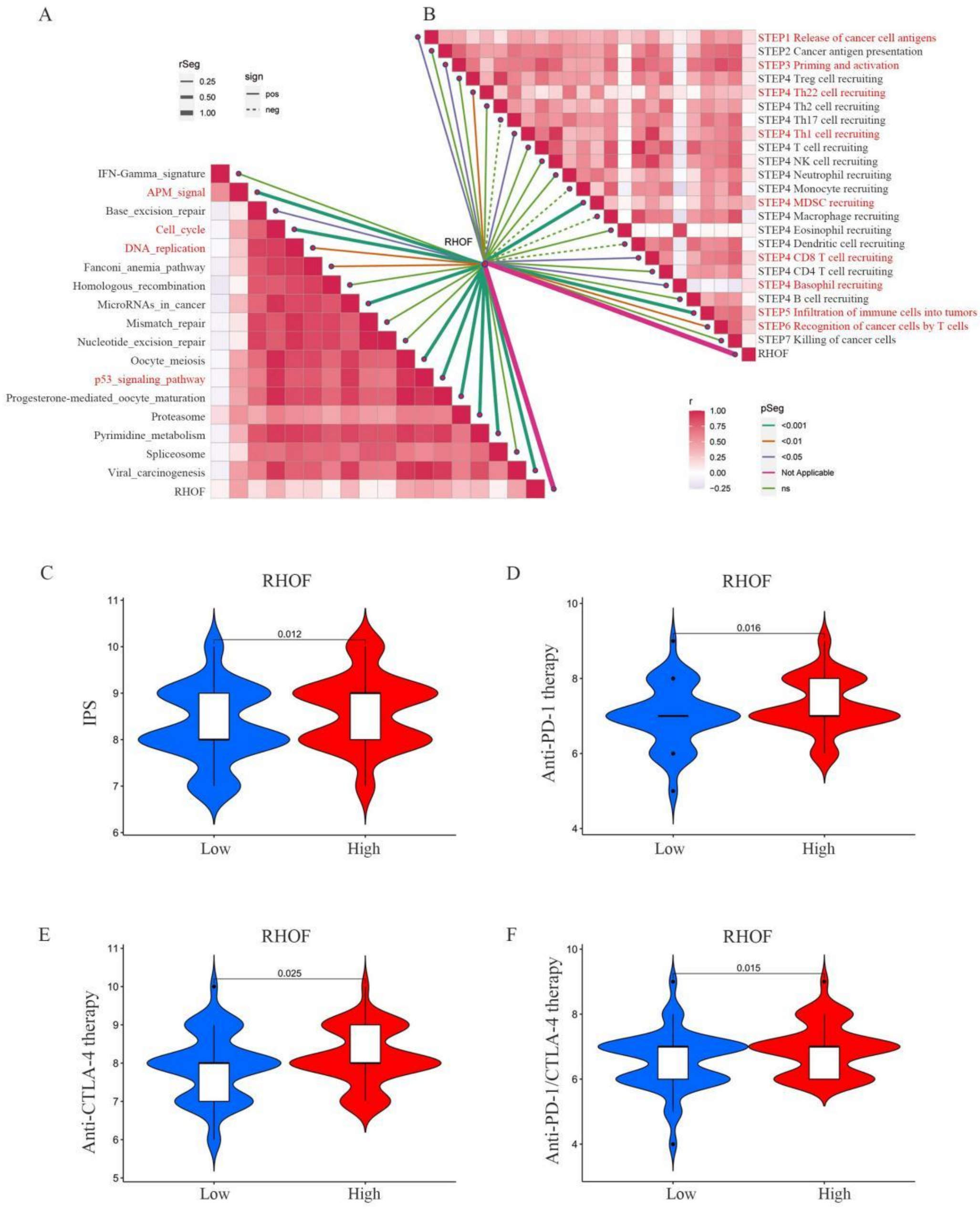

Fig. S3. Correlations between RHOF and enrichment scores of cancer-associated pathways. (A) Correlations between RHOF and the enrichment scores of cancer-associated pathways. (B) Correlations between RHOF and the steps of the cancer immunity cycle. Solid lines represent a positive correlation, dashed lines represents a negative correlation, and the the colors represent significant P-values. (C-F) IPS comparison in low- or high-RHOF groups; (C): IPS; (D) Anti-PD-1 therapy. (E): Anti-CTLA-4 therapy. (F) Anti-PD-1/CTLA-4 therapy(ns: not significant).

Fig. S4. Differential infiltration levels of immune cells between high- and low-IRPM groups. (A-E) The differences in infiltration levels of CD8+ T cells (A), B cells (B), NK cells (C), stromal (D) and microenvironment scores (E) in high- and low-IRPM groups; (F) Response to anti-CTLA-4 immunotherapy of high- and low-IRPM groups in TCGA-PAAD cohort, as assessed by IPS analysis.

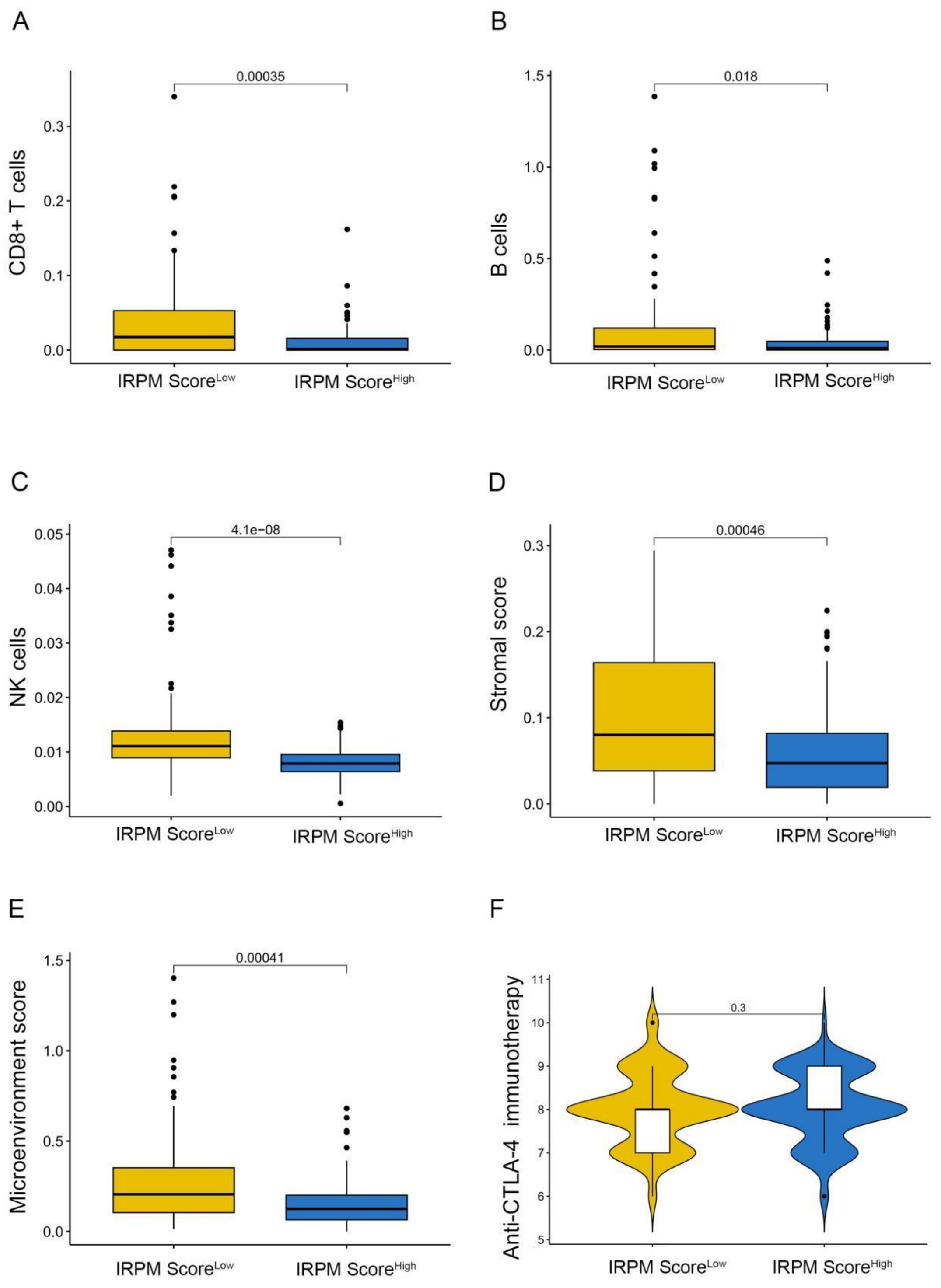

Fig. S5. Single-cell analyses reveal IRPM score heterogeneity in tumor microenvironments. (A) Identify signals contribution of outgoing and incoming signaling pathways by complex heatmap. (B) Heatmaps quantify the role of each cluster as a sender, receiver, mediator, and influencer of TWEAK signaling.

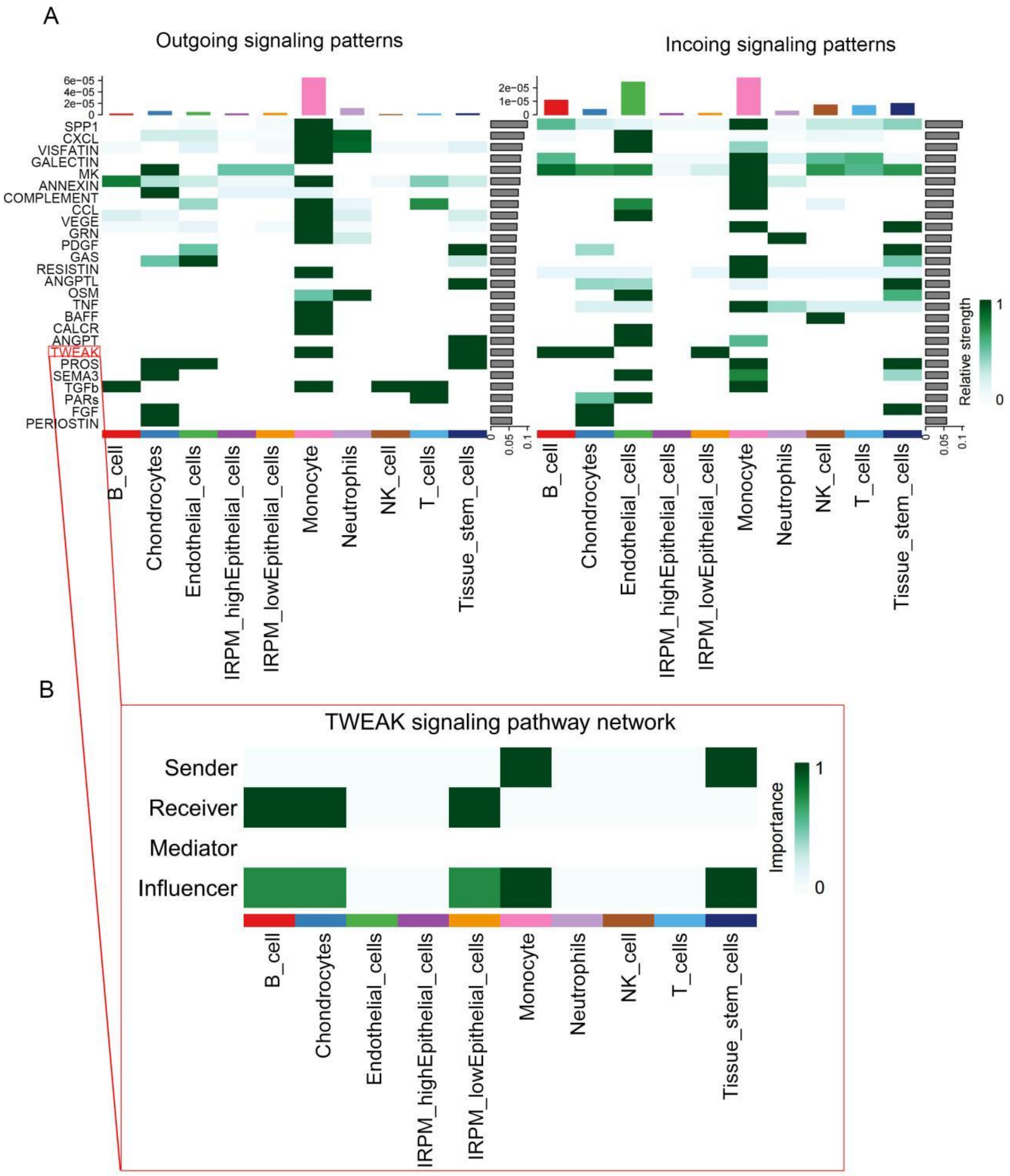

Supplement: Supplementary file 1 [file ijms-25-00142-s001.zip › Supplementary figures.pdf]
