# Supplementary material for: An Integrated Multi-Omics Analysis Identifying Immune Subtypes of Pancreatic Cancer
Source: Int J Mol Sci. 2023 Dec 21;25(1):142. doi: 10.3390/ijms25010142 (PMC10779306; doi:10.3390/ijms25010142)
Supplement: Supplementary file 1 [file ijms-25-00142-s001.zip › Supplementary File S1.pdf]

Also, to further increase the reliability of the results, we reanalyzed our dataset after eliminating the potentially contaminated samples. we have conducted an additional analysis after eliminating the potentially contaminated samples. We reanalyzed the pure pancreatic cancer (PPC) samples (n=150), excluding patients without complete survival information, and ultimately, we included 149 pure pancreatic cancer samples for subsequent analysis. Based on the enrichment scores, 149 PPC patients were divided into three clusters using the non-negative matrix factorization (NMF) method (Fig. R1A-1B); survival analysis revealed that cluster 1 had better prognosis than clusters 2 or 3($P < 0.05$, Fig. R1C). Fig. R1D showed the clustering heatmap for three subtypes and Fig. R1E displays the silhouette width of the NMF clustering, with an average silhouette width of 0.93, indicating that our clustering was stable.

Fig. R2A illustrated the significant differences observed in grade and stage among the cluster groups. Subsequently, we analyzed immune cell infiltration levels among the three clusters by the ssGSEA algorithm (Fig. R2B), and significant distinctions were observed among the three clusters, the cluster 1 showed higher immune cell infiltration, especially for activated B cells, CD8+ T cells, DCs, NK cells and monocyte. The estimated algorithm was further applied to evaluate the TME score in patients with PPC samples. The results also showed that cluster 1, with high estimate (Fig. R2C) and immune scores (Fig. R2D), was correlated with high infiltration levels of immune cells. To further explore roles of the three clusters in immunomodulation, we analyzed the cytokine and chemokine expression of the three clusters: immune activation (IA)-related genes (GZMA, IFNG, TNF, CXCL9, PRF1, GZMB, TBX2, CD8A, and CXCL10)；immune checkpoint (IC)-related genes (PDCD1, LAG3, TNFRSF9, CD86, HAVCR2, CD80, IDO1, TIGIT, CD274 and CTLA4); and TGF)-β/EMT signaling pathway-related genes, including VIM, COL4A1, CLDN3, ACTA2, TGFBR2, ZEB1, and TWIST1. As displayed in Fig. R2E-F, the expression of IA and IC-related genes in cluster 1 was elevated, indicating that the TME exerts a key role in cluster 1. Furthermore, increased levels of TGF-β/EMT signaling pathway-related genes (Fig. R2G) and stromal scores (Fig. R2H) suggest higher degrees of stromal cell infiltration in cluster 1 PPC samples. Moreover, TIDE scores were markedly elevated in the cluster 1 (Fig. R2I). Thus, we concluded that cluster 1 PAAD may stem from tumor-infiltrating immune and stromal cells instead of from cancer cells. Based on the above analysis results, we persistently found our originally proposed clusters. For instance, our original cluster 2 corresponds to the new cluster 1 and both are classified as immune-excluded subtypes. Likewise, our original cluster 3 matches the new cluster 2, both indicating immune-desert subtypes. Moreover, the new cluster 3 is consistent with our original scluster 1, immune-inflamed subtypes. The congruence of the old and new subtypes again displays the robustness of our original classification and reaffirms the credibility of our work.

Furthermore, in order to validate the predictive capacity of our prognostic gene set, we implemented an external validation on 149 pure pancreatic cancer samples which showed consistent results. The performance of our prognostic model is shown to be robust across datasets as indicated by significant differences in survival curves between high- and low-scoring patient groups both in the original dataset and in the external dataset. (Fig. R3A-B). Similarly, the survival curves of also revealed that the risk gene sets, hallmark g2m checkpoint, and thallmark mitotic spindle, with high enrichment scores correlated with poorer prognosisin the PPC data set. (Fig. R3C-D).

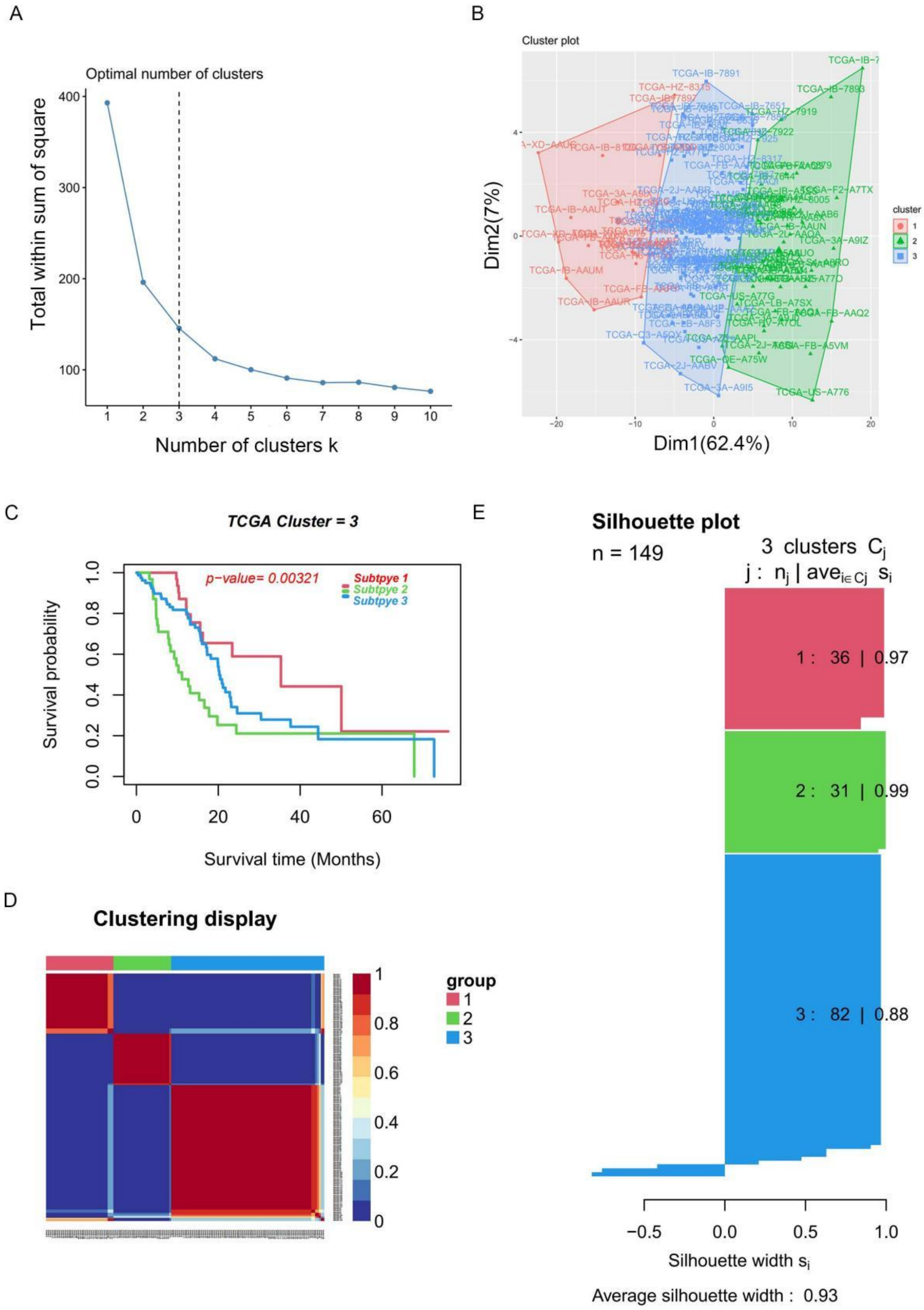

Fig. R1. Identification of PAAD subtypes. (A) K = 3 was the optimal suggested value of number of clusters. (B) Visualisation of the cluster results for TCGA-PAAD(n=149) samples. (C) Survival analysis for PAAD patients among clusters. (D) Consensus map of NMF clustering. (E) Silhouette plot for the PAAD clusters.

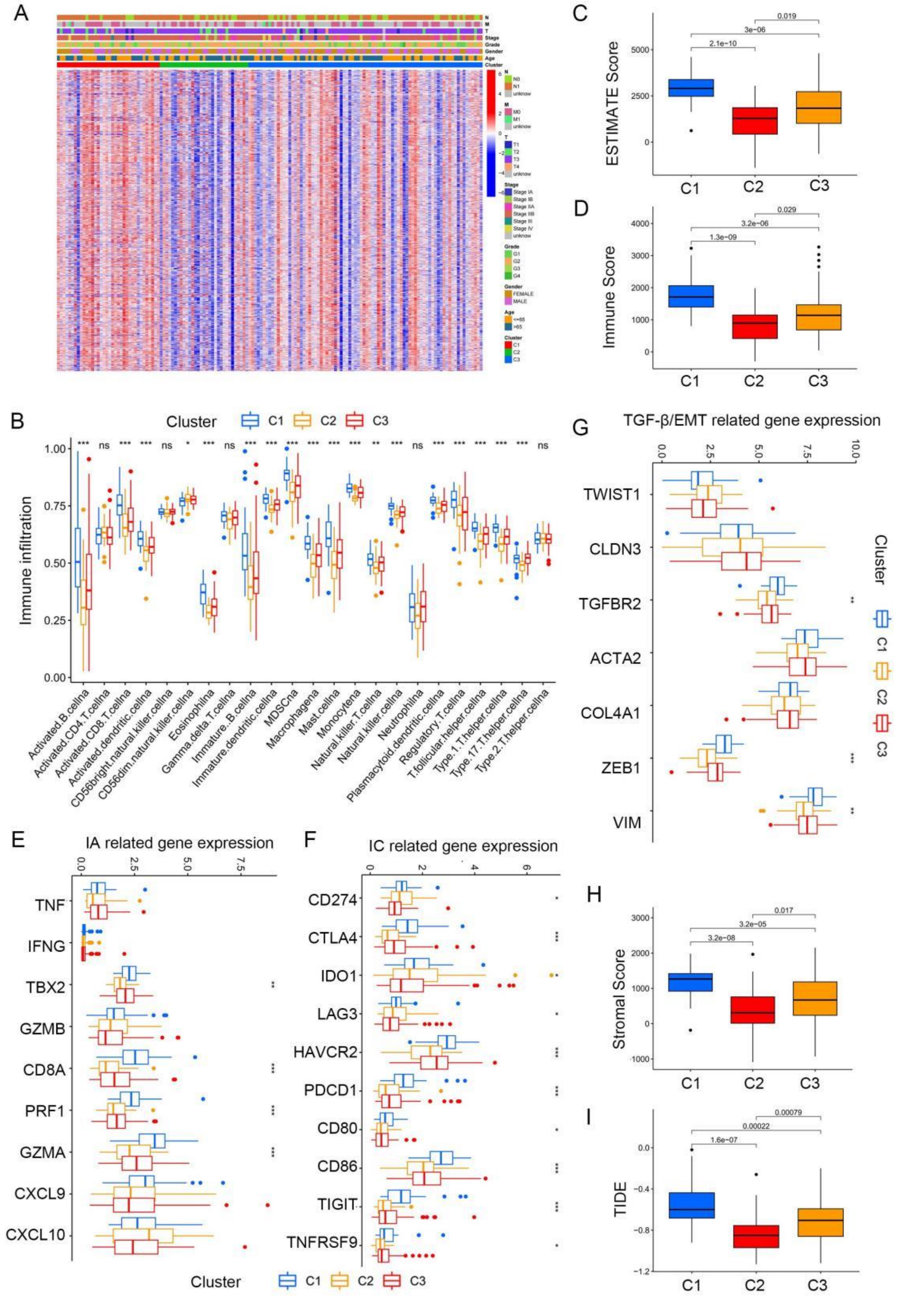

Fig.R2. Immune Landscape in TCGA-PAAD (n=149) patients among three clusters. (A) Heatmap demonstrating the clinicopathologic features among three clusters. (B) The infiltration levels of 23 immune cells in the three clusters. (C-D) The ESTIMATE score(C) and Immunoscore (D) in clusters 1, 2, and 3. (E-G) The expression levels of immune activation (E), immune checkpoint related genes (F) and TGF-β/EMT signaling pathway-related genes (G) in the three clusters. (H-I) Differences in stromal score (H) and TIDE scores (I) in clusters 1, 2, and 3. *p < 0.05, **p < 0.01, and ***p < 0.001.

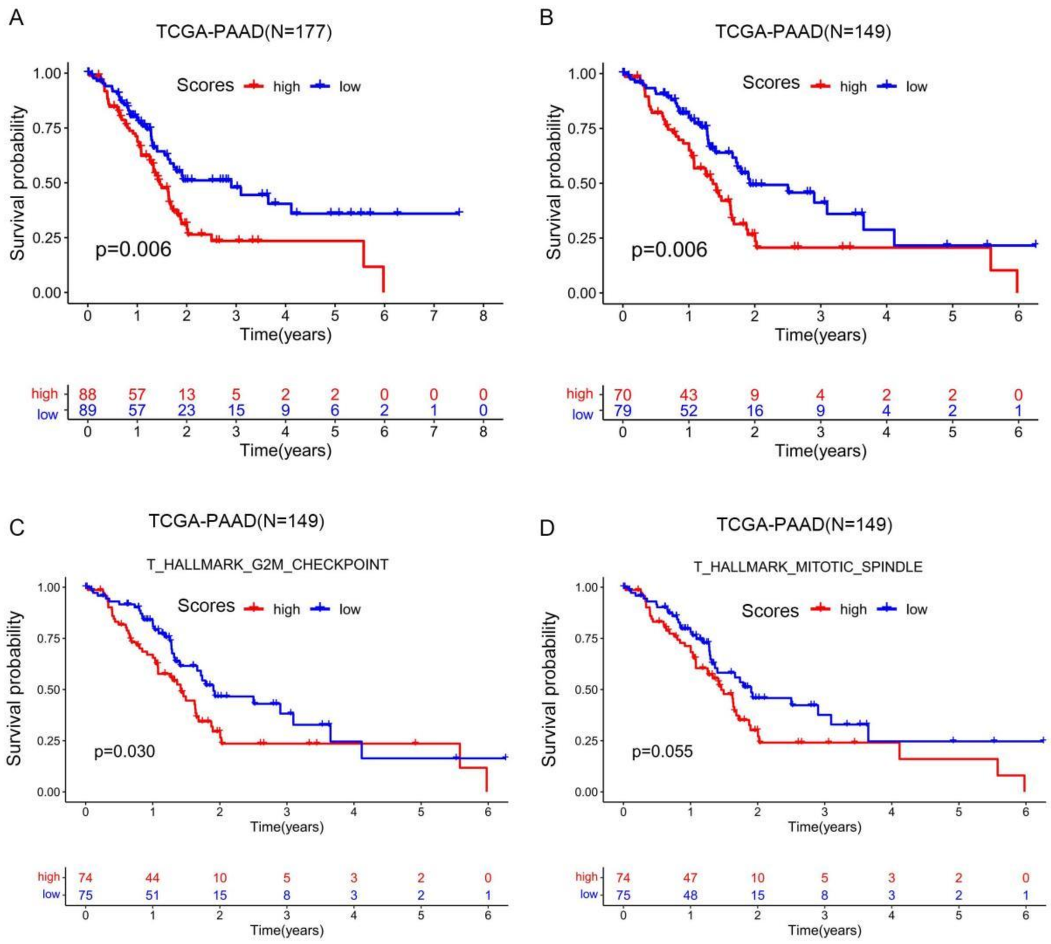

Fig.R3. Prediction performance evaluation of risk scores in the pure pancreatic cancer cohort (n=149). (A-B) Survival analysis for PAAD patients based on high or low enrichment score in TCGA-PAAD (n=177) cohort and PAAD pure pancreatic cancer cohort (n=149). (C-D) Survival analysis for PAAD patients based on high or low gene set enrichment score in TCGA-PAAD pure pancreatic cancer cohort (n=149), C: T_HALLMARK_G2M_CHECKPOINT; D: T_HALLMARK_MITOTIC_SPINDLE.
